# Hypertransaminasemia in hospitalized children: Insights from a national multicenter study by the Italian Society of Pediatric Gastroenterology, Hepatology, and Nutrition

**DOI:** 10.1002/jpn3.70540

**Published:** 2026-08-18

**Authors:** Angelo Di Giorgio, Giusy Ranucci, Pietro Aliberti, Carmela Pia Senatore, Camilla Calì, Angelo Mazza, Francesca Sbravati, Patrizia Alvisi, Maurizio Giuseppe Fuoti, Ilaria Rochira, Antonio Marseglia, Maria Rosa Pastore, Annalisa Madeo, Alessandro La Rosa, Federica Ferrari, Fortunata Sabrina Civitelli, Valentina Maffini, Icilio Dodi, Serena della Femina, Andrea Colangelo, Lorenzo D'Antiga, Claudia Mandato

**Affiliations:** ^1^ Paediatric Hepatology, Gastroenterology and Transplantation, Hospital Papa Giovanni XXIII University of Udine Palermo Italy; ^2^ Pediatric Department IRCCS ISMETT (Istituto Mediterraneo per i Trapianti e Terapie ad alta specializzazione) Palermo Italy; ^3^ UPMC (University of Pittsburgh Medical Center) Italy Palermo Italy; ^4^ Department of Translational Medical Science, Section of Pediatrics University of Naples “Federico II” Naples Italy; ^5^ University of Salerno Fisciano Italy; ^6^ Epidemiology and Biostatistics Santobono‐Pausilipon Pediatric Hospital Naples Italy; ^7^ General Paediatric Department Hospital Papa Giovanni XXIII Bergamo Italy; ^8^ Pediatric Gastroenterology Unit Maggiore Hospital Bologna Italy; ^9^ Pediatric Gastroenterology and Endoscopy Unit, University Department of Pediatrics, Children's Hospital Spedali Civili Brescia Italy; ^10^ Pediatria ‐ IRCCS Casa Sollievo della Sofferenza San Giovanni Rotondo Foggia Italy; ^11^ Pediatric Gastroenterology and Endoscopy Unit IRCCS Istituto Giannina Gaslini Genoa Italy; ^12^ Pediatric Unit Sant'Eugenio Hospital Rome Italy; ^13^ General and Emergency Pediatric Unit ‐ “Pietro Barilla” Children Hospital Parma Italy; ^14^ Department of Health Sciences, Meyer Children's Hospital IRCCS University of Florence Florence Italy; ^15^ Department of Medicine, Surgery and Dentistry “Scuola Medica Salernitana”, Pediatrics Section University of Salerno Salerno Italy; ^16^ Pediatric Hepatology, Gastroenterology and Transplantation Hospital Papa Giovanni XXIII Bergamo Italy; ^17^ Department of Medicine and Surgery University of Milano‐Bicocca Milan Italy; ^18^ Pediatric Chronic Disease, Hepatology and Nutrition Santobono‐Pausilipon Pediatric Hospital, IRCCS Naples Italy

**Keywords:** elevated transaminases, liver disease, pediatric hospitalization

## Abstract

**Objectives:**

Hypertransaminasemia is a frequent finding in hospitalized children with common pediatric illnesses, often considered a transitory phenomenon associated with systemic inflammation/injury. This study aims to assess the prevalence and causes of incidentally detected hypertransaminasemia in children admitted to general pediatric units for non‐chronic liver‐related illnesses.

**Methods:**

This is a prospective, multicenter observational study. Children with transaminase measurement at hospital admission were enrolled from June 2021 to July 2023. Inclusion criteria were: Age >30 days to 17 years; hospitalization for any clinical condition; Informed consent. Patients with a previous diagnosis of liver disease were excluded.

**Results:**

A total of 1652 patients (1 month to 17 years) were enrolled in nine Italian pediatric hospital centers. Overall, the prevalence of hypertransaminasemia was 34.4% (*n* = 570). The majority had mild to moderate liver enzymes elevation. Positive correlation were found between aspartate aminotransferase and creatin P‐kinase (CPK), body mass index *z*‐score and cholesterol. At discharge, 298 of the 570 re‐tested transaminases: 21.4% (64/298) had persistent combined elevation, 13.8% (41/298) isolated alanine aminotransferase, and 37.6% (112/298) isolated elevation. Only 5.70% of the patients (*n* = 30) with elevated transaminase levels (*n* = 526) were eventually diagnosed with liver disease at discharge, whereas the majority of patients (19.9%, *n* = 105) received a diagnosis of pneumonia/bronchitis/upper respiratory tract infection, followed by unspecified infections (10.6%, *n* = 56).

**Conclusions:**

Hypertransaminasemia is common in children admitted to the general pediatric units, affecting approximately 35% of patients. Liver‐related discharge diagnoses were uncommon among children with elevated transaminases (5.70%), whereas most cases occurred in the setting of acute non‐hepatic illnesses. Children with persistently elevated transaminases at discharge should be followed to rule out chronic liver disease.

## Abstract (highlights)

**What is Known:**

- Hypertransaminasemia is relatively common and transient in hospitalized children.
- Hypertransaminasemia may indicate underlying liver disease.

**What is New:**

- Approximately 35% of hospitalized children present with elevated aspartate aminotransferase and alanine aminotransferase, with either isolated or combined elevation.
- Only a minimal percentage of patients with elevated transaminase levels (5.70%) is eventually diagnosed with liver disease.
- The majority resolve hypertransaminasemia during hospitalization.

## 1 INTRODUCTION

Incidental detection of elevated liver enzymes is relatively common in children undergoing routine blood tests for various clinical reasons.^1^, ^2^, ^3^ These alterations are often transient and benign, but persistent abnormalities may raise concern for a possible underlying liver disease, the most frequent being metabolic dysfunction–associated steatotic liver disease (MASLD), infections, genetic or metabolic disorders, autoimmune or endocrine conditions, and drug‐induced liver injury.^2^, ^3^, ^4^, ^5^ Given its high frequency in children with common acute illnesses, distinguishing transitory, self‐resolving hypertransaminasemia from chronic liver disease appears important, to avoid waste of resources on one side but also missing the diagnoses of a chronic, progressive condition on the other side.

In most cases, mild elevations reflect transient cell necrosis associated with systemic inflammation due to common viral infections or to medication exposure, while more rarely, the aspartate aminotransferase, alanine aminotransferase (AST/ALT) increase is persistent, in which case a structured diagnostic approach is warranted. Non‐alcoholic or MASLD has increasingly emerged as the most frequent chronic cause of increased transaminases in older or overweight children, whereas transient viral hepatitis remains predominant in younger ones.^6^

In a large 10‐year single‐center retrospective study, infections accounted for 30% of liver diseases, followed by undiagnosed cases (26%), biliary disorders (16%), and inherited metabolic conditions (14%), while MASLD showed a significant upward trend over the last decade.^7^

In this study, we aimed to investigate the prevalence and clinical significance of incidentally detected hypertransaminasemia in children admitted to general pediatric wards across Italy in a large prospective cohort (Supporting Information S1: Figure S1).

## 2 METHODS

### 2.1 Ethics statement

This study was approved by the local ethics committee at Santobono‐Pausilipon Pediatric Hospital of Naples which was the coordinating center for this study (Prot.N. 00027699).

This was a prospective, multicenter observational study coordinated by the Liver Working Group of the Italian Society for Pediatric Gastroenterology, Hepatology and Nutrition (SIGENP).

### 2.2 Study population

Children admitted to general pediatric wards for a non‐liver‐related illness from June 2021 to July 2023 were enrolled in the study. The sample size was estimated based on data provided by the Italian Ministry of Health. Each center enrolled at least 180 children. No laboratory or other diagnostic tests were performed for research purposes. All investigations were conducted as part of routine clinical care (Supporting Information S1: Table S1).

*Definitions* **:** Hypertransaminasemia was defined as an elevation of AST and/or ALT levels exceeding the standard laboratory reference range.

Hypertransaminasemia was primarily defined using a uniform cutoff of 40 U/L for both AST and ALT, chosen to improve comparability across centers, as this threshold was the most commonly adopted among participating laboratories. Center‐specific reference ranges are reported in Supporting Information S1: Table S2. Prevalence was stratified by sex, hospital level, and age group at admission.

### 2.3 Inclusion criteria

- Patients admitted to general pediatric wards (primary, secondary or tertiary care centers), for whom measurement of transaminase levels was performed.
- Age >30 days to 17 years.
- Children hospitalized for any clinical condition.
- Informed consent provided by patient or legal guardian.

Exclusion criteria were: (i) Hospitalization due to suspected chronic liver disease; (ii) known history of chronic liver disease; (iii) known history of any condition associated with elevated liver enzymes.

### 2.4 Assessments and endpoints

Transaminase levels were measured at admission and subsequently whenever clinically indicated based on the patients’ condition.

Endpoint to determine the prevalence of hypertransaminasemia in children.

### 2.5 Statistical analysis

To calculate the appropriate sample size, we used the Dixon and Massey formula,^8^ based on key assumptions relevant to the study design. Hospital admissions data from year 2017 (diagnosis related groups (DRGs) by the Italian Ministry of Health, the last available at the beginning of the study) were analyzed, excluding surgical and neonatology units, and focusing on the 15 most frequent DRGs stratified by age. A two‐tailed 95% confidence interval (CI) was applied. Using these parameters, NQuery Advisor 7.0 estimated a minimum sample size of 1500 patients to achieve sufficient statistical power. Descriptive statistics were used to analyze demographic and clinical data (mean, median, interquartile range [IQR], and standard deviation for continuous variables; frequencies and percentages for categorical variables). For each parameter, the proportion of valid cases was also calculated. All analyses were performed using R version 4.2.1 (R Core Team, 2022).

## 3 RESULTS

A total of 1652 patients from nine centers (primary *n* = 2; secondary *n* = 4; tertiary *n* = 3) were enrolled in this study. For each patient medical history, demographic and clinical data, diagnostic information, anthropometric parameters, laboratory results, and imaging studies were collected and analyzed (Supporting Information S1: Table S1).

Demographic, clinical, and laboratory data are summarized in Table 1.

**Table 1:** Demographic, clinical, and laboratory features of the study cohort at the time of hospital admission (*n* = 1652 patients) and of the cohort patients showing elevated transaminase levels (AST, ALT, or both) exceeding the laboratory reference values (*n* = 570).

	Entire cohort (*n* = 1652)			Cohort of patients with elevated transaminase levels (AST, ALT, or both) exceeding the laboratory reference values (*n* = 570)		
	Mean (SD)	Median (IQR)	% valid values	Mean (SD)	Median (IQR)	% valid values
Age (months)	66.76 (61.97)	44.00 (14.00–116.00)	99.76	45.18 (54.16)	21.50 (8.00–61.25)	99.65
Duration of hospitalization (days)	7.24 (6.64)	6.00 (4.00–8.00)	44.98	8.01 (8.46)	6.00 (4.00–9.00)	47.54
Weight (kg)	23.21 (18.83)	16.00 (9.80–34.00)	97.88	17.44 (16.73)	11.50 (7.99–18.90)	98.77
Weight (*z*‐score)	0.03 (1.75)	0.12 (−0.75 to 1.04)	57.33	−0.11 (1.74)	−0.01 (−0.95–0.93)	55.61
Height (cm)	107.69 (34.75)	103.00 (79.67–137.12])	47.94	92.20 (30.36)	85.00 (72.00–105.50)	45.79
Height (*z*‐score)	0.27 (1.52)	0.30 (−0.50 to 1.11)	43.10	−0.56 (11.98)	0.06 (−0.70 to 1.04)	45.79
BMI	18.16 (4.99)	17 (14.9–19.92)	27.48	16.92 (4.45)	15.55 (14.27–18.10)	79.64
BMI (*z*‐score)	−0.07 (1.85)	0.1 [−0.96 to 1.15]	31.00	−0.47 (2.08)	−0.30 (−1.58 to 1.04)	20.35
Weight/Height (*z*‐score)	0.17 (2.28)	0.03 (−1.06 to 1.09)	16.72	0.18 (2.36)	0.02 (−1.19 to 0.99)	25.44
Hb (g/dL)	12.26 (1.60)	12.40 (11.30–13.30)	37.83	11.95 (1.68)	12.00 (10.90–13.10)	64.56
AST (IU/L)	50.45 (100.79)	33.00 (24.00–45.00)	95.40	93.04 (159.33)	51.00 (43.00–68.75)	99.30
ALT (IU/L)	39.12 (99.94)	20.00 (14.00–29.00)	99.94	79.48 (189.62)	31.00 (21.00–57.00)	100.00
GGT (U/L)	34.35 (28.21)	15.00 (12.00–23.75)	28.21	57.80 (103.95)	21.00 (13.25–54.25)	34.04
HDL (mg/dL)	41.46 (18.96)	37.00 (27.00– 53.00)	4.90	37.66 (17.65)	35.00 (24.50–47.75)	10.18
LDL (mg/dL)	92.53 (49.59)	83.00 (61.50–111.00)	4.36	88.42 (45.07)	77.50 (60.00–110.25)	8.77
Triglycerides (mg/dL)	103.65 (107.41)	83.00 (56.00–120.50)	18.89	131.19 (142.54)	101.00 (76.50–149.75)	27.02
Total cholesterol (mg/dL)	149.29 (45.15)	143.00 (120.25–168.50)	9.08	148.19 (49.86)	142.00 (115.00–174.00)	17.02
Bile acids (µmol/L)	38.84 (38.46)	24.00 (14.45–57.50)	0.42	38.84 (38.46)	24.00 (14.45–57.50)	1.23
Unconjugated bilirubin (mg/dL)	0.45 (1.32)	0.21 (0.16–0.39)	30.63	0.56 (1.69)	0.25 (0.16–0.47)	51.93
Conjugated bilirubin (mg/dL)	0.27 (0.62)	0.17 (0.10–0.24)	30.99	0.31 (0.79)	0.10 (0.10–0.22)	51.93
LDH (IU/L)	422.14 (539.81)	316.00 (233.25–491.25)	31.60	541.73 (673.54)	435.00 (320.00–611.00)	53.51
CPK (IU/L)	508.94 (4387.61)	94.50 (63.25–163.75)	27.24	773.86 (5641.99)	101.00 (60.00–193.50)	47.54

Abbreviations: ALT, alanine aminotransferase; AST, aspartate aminotransferase; BMI, body mass index; CPK, creatin P‐kinase; GGT, gamma‐glutamyl transferase; Hb, haemoglobin; HDL, high‐density lipoprotein; IQR, interquartile range; LDH, lactate dehydrogenase; LDL, low‐density lipoprotein; SD, standard deviation.

The mean age at hospitalization was 66.7 months (around 5 and a half years old), with a standard deviation of 61.9 months, indicating a wide dispersion of values around the mean. The median age was 44 months (IQR: 14–116), reflecting a skewed distribution with a higher prevalence of younger patients.

Transaminase values had non‐normal distribution with marked positive skewness, meaning a long right tail (Table 1, Supporting Information S1: Figure S2). This pattern indicates the presence of outliers in a minority of patients. Mean and median ALT values were 39.1 U/L (±99.4) and 20 U/L (IQR: 14–29) respectively. Similarly, mean and median AST values were 50.4 U/L (±100.7) and 33 U/L (IQR: 24–45) respectively, confirming the wide variability and skewness in the data.

The very high standard deviation (99.9 for ALT and 100.7 for AST) suggests that the median and interquartile range are more appropriate indicators to describe the distributions of these variables.

Among the 1652 patients enrolled in our study there was a slight predominance of males (53.3%), which did not represent a significant imbalance. Regarding age distribution, the majority of patients were in the 0–3‐year age group, indicating a higher representation of younger children in the cohort.

Concerning the distribution by hospital level of specialization, the largest proportion of patients comes from secondary level centers (47.15%).

Supporting Information S1: Table S3 highlights key clinical and demographic differences between sexes. While mean AST was higher in females, males had a higher median value, suggesting greater variability in the female group. Creatin P‐kinase (CPK) levels were significantly higher in males (579.9 vs. 439.3 U/L, *p* < 0.001), consistent with known sex‐related differences in muscle enzyme profiles.

### 3.1 Patients with hypertransaminasemia

Table 2, Supporting Information S1: Table S4 and Supporting Information S1: Figure S3 show the prevalence of hypertransaminasemia by age, gender, and type of referral center. Overall, 34.5% (*n* = 570) of patients had elevated transaminases: 12.1% (*n* = 199) showed combined ALT and AST elevation, 2.06% (*n* = 34) with isolated ALT elevation, and 20.3% (*n* = 335) with isolated AST elevation. Among these, ALT values were ≤100 U/L in 87%, 100–200 U/L in 6%, and >200 U/L in 7%; AST values were ≤100 U/L in 84%, 100–200 U/L in 8%, and >200 U/L in 8%. No sex differences emerged. Children aged 0–1 years had the highest rate of combined elevation (*p* < 0.001); isolated AST was more frequent in ages 1–3, and isolated ALT in >11 years (*p* < 0.05). Of the 570 patients with elevated transaminases at admission, only 298 (52.3%) were re‐tested at discharge: 21.4% (64/298) had persistent combined elevation, 13.8% (41/298) isolated ALT, and 37.6% (112/298) isolated elevation.

**Table 2:** Prevalence of ALT and AST stratified by age (in years).

Prevalence	All patients (*n* = 1652)	Age at hospitalization (in months) divided into groups					
		0–1 (*n* = 363)	1–3 (*n* = 372)	3–6 (*n* = 291)	6–11 (*n* = 276)	>11 (*n* = 346)	*p*‐value
ALT and AST high (%)	12.17	19,23	11.56	7.90	10.14	10.4	<0.001***
Only ALT high (%)	2.06	1.65	1.34	0.69	2.9	3.76	0.04*
Only AST high (%)	20.28	29.12	34.68	18.21	10.14	5.2	<0.001***
None high (%)	65.60	50	52.42	73.2	76.81	80.64	<0.001***

*Note*: The laboratory reference values are considered as cutoff points. The values in the table are expressed in percentages. It covers all the patients. * and *** represent the significance at levels 5% and 0.1%, respectively.

Abbreviations: ALT, alanine aminotransferase; AST, aspartate aminotransferase.

Because age‐ and sex‐related differences may influence transaminase interpretation in childhood, an additional subanalysis using the 97th percentile thresholds proposed by Bussler et al. was performed (Supporting Information S1: Table S5). This showed substantial variation in the distribution of isolated AST and ALT abnormalities according to the cutoff applied, confirming that prevalence estimates are strongly dependent on the definition used.

### 3.2 Evaluation of transaminase at discharge

Among the 298 patients who underwent repeat evaluation of liver enzymes, normalization of values was observed 27.2% (*n* = 81) of cases after discharge, while 72.8% (*n* = 217) of patients still had elevated transaminase levels.

Interestingly, patients with elevated transaminases were significantly younger (median age 1 year) and had lower weight *z*‐scores and body mass index (BMI). (Supporting Information S1: Figure S4).

### 3.3 Correlation between AST, ALT, and other parameters

Figure 1 shows the correlations between AST, ALT, GGT (gamma glutamyl transferase), and other clinical parameters in the studied population. A strong positive correlation is observed between AST and CPK, suggesting that in many patients AST was of muscular origin, as well as between BMI *z*‐score and cholesterol.

**Figure 1:**
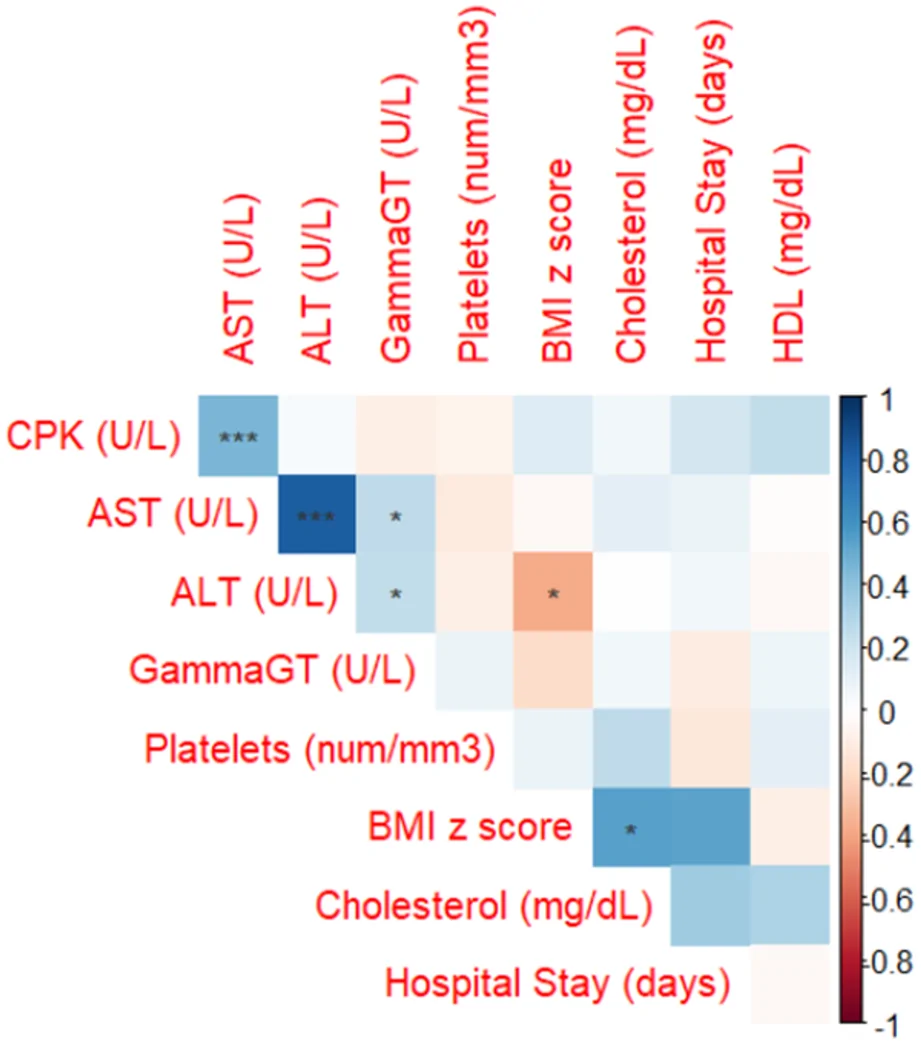
Correlation plot between ALT, AST, GGT, CPK, Duration of the hospitalization, BMI *z*‐score, platelet and cholesterol for patients above laboratory cutoff. *, **, and *** represent the significance at levels 5%, 1%, 0.1%, respectively. ALT, alanine aminotransferase; AST, aspartate aminotransferase; BMI, body mass index; CPK, Creatin P‐kinase; GGT, gamma glutamyl transferase; HDL, high‐density lipoprotein.

### 3.4 Diagnostic group

Given the large number of diagnoses and ICD‐9‐CM codes, these were grouped into Diagnostic Groups to facilitate analysis (Supporting Information S1: Table S6).

The most frequent diagnoses of patients with elevated transaminase levels are shown in Table 3. Liver disease diagnoses were found to be very rare across the entire study cohort. At hospital admission, only 2.63% of patients with hypertransaminasemia received a diagnosis related to liver diseases, while at discharge this percentage slightly increased to 5.70% (Table 3). Patients discharged with the label “elevated transaminases” (*n* = 17/525, 3.23%) did not have an identified etiological diagnosis and were not considered as confirmed liver disease.

**Table 3:** Most frequent diagnosis and diagnosis of liver disease in patients with elevated transaminase level.

	Admission		Discharge	
**Diagnosis**	**Number of patients**	**% of the cohort with hypertransaminasemia (*n* = 570)**	**Number of patients**	**% of the cohort with hypertransaminasemia (*n* = 526)**
Most frequent diagnosis in patients with elevated transaminase level				
Pneumonia/Bronchitis/upper respiratory tract infection	105	18.4	105	19.9
Infections (unspecified)	40	7.0	56	10.6
Infectious gastroenteritis	68	11.9	48	9.1
Seizures	51	8.9	41	7.79
Trauma	35	6.1	35	6.6
Fever	73	12.8	18	3.4
Diagnosis of liver diseases in the cohort				
Acute hepatitis	8	1.40	8	1.52
Non alcoholic fatty liver disease	3	0.52	5	0.95
Acute liver failure			1	0.19
Liver malformations	3	0.52	11	2.09
Biliary atresia	1	0.17	5	0.95
Total	15	2.63	30	5.70

Across the entire cohort (*n* = 1652), 73 patients presented with elevated AST and CPK levels at admission. Among these, discharge diagnoses were available for 63 patients: 50.79% (*n* = 32) received a diagnosis of infection, 12.69% (*n* = 8) a diagnosis of seizures, 7.9% (*n* = 5) a diagnosis of trauma, and 4.76% (*n* = 3) a diagnosis of cholestasis, while 9.52% (*n* = 6) received other diagnoses.

## 4 DISCUSSION

In this prospective multicenter study, we assessed the prevalence and clinical relevance of hypertransaminasemia in a large cohort of hospitalized children. About 35% presented with elevated transaminases during hospitalization, confirming its common occurrence in pediatric inpatients. Prevalence varies widely: Nobili et al. found ALT elevations in 27.4% of 3280 emergency‐unit encounters, with 20% persisting at follow‐up^9^; in a Swedish obesity registry, 37.9% had mildly and 10.6% markedly elevated ALT^10^; conversely, in a multicenter study of adenovirus respiratory infections, ALT was elevated in 7.7% but AST in 46.9% of cases.^11^

Most patients with hypertransaminasemia had mild to moderate elevations, with AST and ALT < 100 U/L.

When looking at age‐related differences, ALT levels present the highest elevation in infants aged 0–1 years, whereas AST in children aged 1–3.

First‐level hospitals usually admit younger children, in whom AST is physiologically higher, as also reported by Bussler et al.^12^ and Schwimmer et al.^13^ This highlights need for cautious interpretation of hypertransaminasemia in younger patients, where clinical assessment, neurological exam, and CPK testing can help distinguish hepatic from muscular or viral causes. Tertiary centers showed more frequent combined AST/ALT elevations, likely reflecting referral of more complex, often older patients, consistent with the influence of age and comorbidities on transaminase variability.^4^, ^14^

Elevated liver enzymes have been associated with higher mortality in children hospitalized for severe community‐acquired pneumonia,^15^ and increased AST in respiratory syncytial virus (RSV) bronchiolitis correlates with worse outcomes.^14^ Viral gastroenteritis, mainly from rotavirus, norovirus, or adenovirus, can also cause transient transaminase elevation^16^ and should be included in the differential diagnosis. Only a small proportion of our patients received a confirmed liver disease diagnosis, with most cases attributed to acute viral infections with systemic inflammation, confirming its role as the leading cause of acute pediatric liver disease.^7^, ^17^

While hypertransaminasemia is common, true hepatopathies are rare; thus, elevated transaminases require careful clinical assessment, including viral screening when indicated, without assuming hepatic origin. In addition, a proportion of patients did not receive an etiological diagnosis and were classified as non‐specific hypertransaminasemia, reflecting the real‐world nature of the study. In our cohort, higher AST was linked to longer hospital stay. Given its lower specificity for liver disease, this may reflect systemic conditions, infections, hemolysis or myopathies. In the context of an acute infectious episode, benign acute children myositis could be a possible cause of elevated CPK levels.^18^ The positive AST–CPK correlation suggests that, in many cases, AST elevation reflected a muscular rather than a liver injury. This supports CPK testing in isolated hypertransaminasemia, even in neurologically asymptomatic children, to exclude myopathies.^19^

Interestingly, ALT and GGT levels showed a negative association with BMI z‐score, contrasting with the well‐established relationship between obesity and MASLD reported in population‐based studies.^12^, ^20^, ^21^ Indeed, both European Society for Pediatric Gastroenterology, Hepatology and Nutrition (ESPGHAN) and North American Society for Pediatric Gastroenterology, Hepatology and Nutrition (NASPGHAN) recommend routine ALT screening in overweight and obese children for the detection of steatotic liver disease,^22^ and Johansen et al. confirmed this association in a cohort of Danish children, supporting the link between obesity and non‐alcoholic fatty liver disease (NAFLD)/MASLD.^23^

The inverse association observed in our study could be related to the younger age of our hospitalized cohort and to the clinical characteristics of children requiring inpatient care. In particular, younger children and those with lower BMI *z*‐scores may be more prone to hospitalization for acute or intercurrent conditions associated with transient hypertransaminasemia. Consistently, most patients who were retested at discharge showed normalization of transaminase levels, suggesting that their elevations are often transient and self‐limiting in the general hospitalized pediatric population rather than being primarily driven by obesity‐related liver disease.^24^

Scheduled outpatient follow‐up should be recommended to patients with unexplained, mild hypertransaminasemia^25^ in order to avoid missed cases of chronic liver disease, which may progress silently to cirrhosis if undiagnosed.^17^, ^26^, ^27^

This study has some limitations. One of the main limitations of the study is the definition of hypertransaminasemia using a uniform cutoff across centers. This approach was adopted to improve comparability in a multicentre real‐world setting, but it does not account for inter‐laboratory variability, nor for the known age‐ and sex‐related differences in pediatric AST and ALT values. Sensitivity analyses using percentile‐based thresholds showed that prevalence estimates and the distribution of isolated AST or ALT elevation vary according to the cutoff applied. Therefore, our prevalence findings should be interpreted as pragmatic real‐world estimates rather than universally applicable epidemiological thresholds. The high proportion of younger children in our cohort did not allow the use of National Health and Nutrition Examination Survey (NHANES)‐based ALT cutoffs (26 U/L in boys and 22 U/L in girls) as an alternative to the traditional 40 U/L threshold. Indeed, these cutoffs were primarily derived for children and adolescents aged 8–17 years and are not well established for children younger than 8 years, particularly infants. In many cases, no definitive etiological diagnosis was reached, variability in laboratory methods and data collection across centers may have influenced results, and the potential hepatotoxic effects of treatments administered during hospitalization were not evaluated. Moreover, the young age of the studied cohort could be considered a further limitation because the majority of patients with undisclosed liver disease is usually older. The strengths are: (i) the large, unselected pediatric cohort, improving the generalizability of results to routine clinical practice; (ii) the multicenter design, capturing a broad age range and levels of care, enhancing representativeness.

## 5 CONCLUSION

In conclusion, hypertransaminasemia is a common finding in hospitalized children and is most frequently associated with acute systemic or non‐hepatic conditions, particularly infections. In most cases, transaminase elevations are mild to moderate and likely transient, whereas chronic liver disease appears to be relatively uncommon in this clinical setting. Nevertheless, persistent or progressive abnormalities should prompt appropriate follow‐up and further diagnostic evaluation.

## CONFLICT OF INTEREST STATEMENT

The authors declare no conflicts of interest.

## Supporting information

Supplementary Material REV.
