# Supplementary material for: Hypertransaminasemia in hospitalized children: Insights from a national multicenter study by the Italian Society of Pediatric Gastroenterology, Hepatology, and Nutrition
Source: J Pediatr Gastroenterol Nutr. 2026 Aug 18;83(5):841–8. doi: 10.1002/jpn3.70540 (PMC13736150; doi:10.1002/jpn3.70540)
Supplement: Supplementary file 1 [file JPN3-83-841-s001.docx]

**Hypertransaminasemia in Hospitalized Children. Insights from a National Multicenter Study by SIGENP**

**SUPPELEMENTARY MATERIAL**

***Supplementary figure 1*.** *Distribution of the Hospital Centres involved in the study.*

***Supplementary figure 2****. Histograms of AST, ALT and GGT. The dash lines represent the mean of each variable.*

| **Demographic and clinical data** | **Diagnostic data** | **Auxological Parameters** | **Clinical Data** | **Laboratory Data** | **Imaging studies** |
| --- | --- | --- | --- | --- | --- |
| Age | Admission diagnosis | Weight in kg at admission | Hepatomegaly (Yes/No) | Red blood cells, Haemoglobin and Mean Cell Volume | Abdominal US |
| Sex | Discharge diagnosis | Weight  z-score at admission | Pale Stools (Yes/No) | Platelets | Magnetic Resonance Imaging |
| Age at admission | ICD-9-CM code associated with the diagnosis | Height in cm at admission | Scratch Lesions (Yes/No) | White Blood Cells (Neutrophils, Lymphocytes, Monocytes) | Computed Tomography |
| Month of admission | Diagnostic group | Height  z-score at admission | Hepatic Steatosis (Mild/ Moderate/ Severe/Not present) | Immunology and Autoimmunity Tests (IgG, IgA, IgM, Coombs Test and Celiac test) |  |
| Duration of hospitalization |  | BMI | Liver Cysts (Yes/No) | Liver Function Test (AST, ALT, GGT, direct and indirect bilirubin, bile acids, LDH) |  |
|  |  |  | Celiac Disease (Yes/No) | Lipid Profile (HDL, LDL, Triglycerides, Cholesterol) |  |
|  |  |  | Drugs (Yes/No) | Metabolic and Nutritional Panel (Vitamin D, Calcium, Phosphorus, Magnesium, Ferritin, Serum iron) |  |
|  |  |  |  | Muscle Enzymes (CPK, lactic acid) |  |
|  |  |  |  | Coagulation Panel (INR) |  |
|  |  |  |  | Blood Gas Analysis |  |

***Supplemental table 1****. Data collected from patients enrolled in the study. BMI: Body Mass Index, IgA: Immunoglobulin A, IgG: Immunoglobulin G, IgM: Immunoglobulin M, ALT: Alanine Aminotransferase, AST: Aspartate Aminotransferase, GGT: Gamma-Glutamyl Transferase, HDL: high density lipoprotein, LDL: low density lipoprotein, LDH: Lactate Dehydrogenase, CPK: Creatine Kinase; INR: international normalized ratio.*

| CENTER | CUT-OFF |
| --- | --- |
| Center 1 | ALT 40 AST 40 |
| Center 2 | ALT 45 AST 45 |
| Center 3 | ALT 43 AST 30 |
| Center 4 | ALT 40 AST 40 |
| Center 5 | ALT 40 AST 40 |
| Center 6 | AST 41 ALT 35 |
| Center 7 | ALT 40 AST 40 |
| Center 8 | ALT 40 AST 40 |
| Center 9 | ALT 40 AST 40 |

***Supplemental table 2****. Transaminase cut-off values for each center*

|  | Male (*n*=880 - 53.27%) | | | | Female (*n*=772 – 46.73%) | | | | T-Test  p-value |
| --- | --- | --- | --- | --- | --- | --- | --- | --- | --- |
|  | Mean | Median | IQR | Std Dev. | Mean | Median | IQR | Std Dev. |  |
| **Age (months)** | 63.39 | 43.00 | [12 - 109] | 58.99 | 70.46 | 45.00 | [16.00 – 121.00] | 64.89 | * |
| **Duration of hospitalization (days)** | 7.14 | 5.00 | [3 - 8] | 6.96 | 7.37 | 6.00 | [4.00 - 8.00] | 6.24 |  |
| **Weight (Kg)** | 22.45 | 15.70 | ([9.70 – 32.00]) | 18.28 | 24.09 | 16.00 | [10.00 – 37.05] | 19.42 |  |
| **Weight (*z*-score)** | 0.07 | 0.21 | ([-0.78 – 1.08]) | 1.70 | -0.02 | -0.08 | [-0.72 – 0.98] | 1.80 |  |
| **Height (cm)** | 105.62 | 100.00 | ([77.50 – 134.30]) | 35.34 | 109.97 | 105.80 | [82.00 - 142.00] | 33.98 |  |
| **Height (*z*-score)** | 0.40 | 0.40 | ([-0.45 – 1.30]) | 1.44 | 0.14 | 0.20 | [-0.54 – 0.97] | 1.60 | * |
| **BMI** | 17.71 | 16.70 | ([14.70 – 19.10]) | 4.58 | 18.60 | 17.30 | [15.10 – 20.64] | 5.34 | * |
| **BMI (z-score)** | -0.22 | -0.24 | ([-1.08 – 1.14] | 1.90 | 0.07 | 0.21 | ([-0.84 – 1.16]) | 1.80 |  |
| **Weight/Height (z-score)** | 0.35 | 0.14 | ([-0.98 – 1.28]) | 2.50 | -0.08 | -0.17 | ([-1.26 – 0.83]) | 1.94 |  |
| **AST (IU/L)(n.v.)** | 49.25 | 34.00 | ([26.00 - 46.00]) | 86.16 | 51.84 | 32.00 | [23.00 - 44.00] | 115.46 | *** |
| **ALT (IU/L) (n.v.)** | 38.11 | 20.00 | ([14.00 - 30.00]) | 100.57 | 40.26 | 20.00 | [14.00 - 27.00] | 130.13 |  |
| **GGT (IU/L) (n.v.)** | 35.48 | 16.00 | ([12.00 - 26.00]) | 69.68 | 33.24 | 15.00 | [12.00 - 21.00] | 72.63 |  |
| **Bile acids (mcmol/L) (n.v.)** | 38.67 | 34.00 | [17.50 – 57.50] | 40.20 | 38.97 | 23.00 | [18.23 – 43.75] | 43.36 |  |
| **Unconjugatedbilirubin (mg/dL) (n.v.)** | 0.44 | 0.20 | [0.10 – 0.35] | 0.94 | 0.47 | 0.23 | [0.20 – 0.40] | 1.62 |  |
| **Conjugated bilirubin (mg/dL) (n.v.)** | 0.24 | 0.16 | [0.10 – 0.25] | 0.42 | 0.30 | 0.17 | [0.10 – 0.24] | 0.76 |  |
| **Total Cholesterol (mg/dl) (n.v.)** | 149.70 | 143.00 | [120.00 - 169.00] | 48.63 | 148.90 | 143.00 | [124.00 - 167.00] | 41.90 |  |
| **HDL(mg/dl) (n.v.)** | 43.62 | 38.50 | ([27.75 – 62.25]) | 22.14 | 39.34 | 36.00 | [27.00 - 53.00] | 15.22 |  |
| **LDL (mg/dl) (n.v.)** | 92.55 | 75.50 | ([57.75 – 111.25]) | 53.96 | 92.50 | 86.50 | [63.75 -108.75] | 45.57 |  |
| **Triglycerides (mg/dl) (n.v.)** | 107.86 | 86.00 | ([55.00 - 129.00]) | 136.89 | 99.44 | 78.50 | [57.75 – 117.25] | 66.14 |  |
| **LDH (IU/L) (n.v.)** | 406.10 | 324.00 | [239.00 – 490.50] | 244.82 | 437.93 | 313.00 | [225.00 – 489.50] | 721.05 |  |
| **CPK (IU/L) (n.v.)** | 579.87 | 106.00 | [68.00 – 171.50] | 4606.62 | 439.26 | 84.00 | [58.00 - 180.00] | 4170.30 | *** |
| **Hb (g/dL) (n.v.)** | 12.16 | 12.20 | ([11.10 – 13.30]) | 1.61 | 12.36 | 12.60 | [11.60 – 13.40] | 1.59 | * |
|  | | | | | | | | | |
| **Age at the hospitalization (in years)** | % |  |  |  | % |  |  |  |  |
| **Until 1 years** | 24.06 |  |  |  | 19.71 |  |  |  | * |
| **From 1 to 3 years** | 21.32 |  |  |  | 23.99 |  |  |  |  |
| **From 3 to 6 years** | 18.13 |  |  |  | 17.12 |  |  |  |  |
| **From 6 to 11 years** | 17.56 |  |  |  | 15.82 |  |  |  |  |
| **Greater than 11 years** | 18.93 |  |  |  | 23.35 |  |  |  |  |
|  | | | | | | | | | |
| **Hospitals – Levels of Specialization** |  |  |  |  |  |  |  |  |  |
| **Level 1** | 24.43 |  |  |  | 22.02 |  |  |  |  |
| **Level 2** | 46.59 |  |  |  | 47.80 |  |  |  |  |
| **Level 3** | 28.98 |  |  |  | 30.18 |  |  |  |  |

***Supplementary Table 3.*** *Demographic and Clinical Characteristics of the Study Cohort by Gender. *, ** and *** represent the significance at levels 5%, 1%, 0.01%, respectively.*

| Prevalence | All patients  (*n*=1,652) | Gender | | |
| --- | --- | --- | --- | --- |
|  |  | Male  (*n*=880) | Female (*n*=772) | p-value |
| ALT & AST high (%) | 12.17 | 13.07 | 11.14 | 0.26 |
| Only ALT High (%) | 2.06 | 2.5 | 1.55 | 0.23 |
| Only AST High (%) | 20.28 | 20.91 | 19.56 | 0.53 |
| None high (%) | 65.60 | 63.52 | 67.75 | 0.08 |

***Supplemental Table 4.*** *Prevalence of ALT and AST stratified by Gender. The laboratory reference values are considered as cutoff points. The values in the table are expressed in percentages. It covers all the patients. *, ** and *** represent the significance at levels 5%, 1%, 0.1%, respectively*

| Prevalence (*n*=1,652) | PREVALENCE USING 97th PERCENTILE AS CUT-OFF | PREVALENCE USING LABORATORY REFERENCES AS CUT-OFF |
| --- | --- | --- |
|  |  |  |
| ALT & AST high | 11.20 | 12.17 |
| Only ALT High | 10.35 | 2.06 |
| Only AST High | 3.87 | 20.28 |
| None high | 74.57 | 65.60 |

***Supplemental table 5****. Prevalence of ALT and AST. The Laboratory references and 0.97 percentile is considered as cutoff points. The values in the table are expressed in percentages. It coveres all the patients.*

***Supplementary Figure 3.*** *a) Barplots of prevalence by sex for the entire cohort based on laboratory cut-off. b) Barplots of prevalence by centre for the entire based on laboratory cut-off.*

***Supplementary Figure 4****.* *Distribution of clinical variables (age, weight Z-score at admission, BMI, and hospital stay duration) according to transaminase group classification based on laboratory cut-off. Patients were categorized based on serum transaminase levels as either within normal limits (“None”) or above the laboratory reference cut-off, “Elevated Transaminases”.*

| **DIAGNOSTIC GROUP** | **CODE** | **ICD-9-CM** | **NOTE** |
| --- | --- | --- | --- |
| LIVER TRANSPLANT | V427 | Organ or tissue replaced by transplant - Liver |  |
| CHRONIC HEPATITIS | 57140 | Chronic hepatitis, unspecified |  |
|  | 57141 | Chronic persistent hepatitis |  |
| ACUTE HEPATITIS | 07053 | Acute hepatitis E without hepatic coma |  |
|  | 0701 | Hepatitis A without hepatic coma |  |
|  | 0709 | Unspecified viral hepatitis without mention of hepatic coma |  |
|  | 07059 | Other specified viral hepatitis without mention of hepatic coma |  |
|  | 5731 | Hepatitis in classified viral diseases |  |
|  | 5732 | Hepatitis in other infectious diseases |  |
|  | 5733 | Hepatitis, unspecified |  |
| NON ALCOHOLIC FATTY LIVER DISEASE | 5718 | Other chronic non-alcoholic liver disease |  |
| ACUTE LIVER FAILURE | 570 | Acute and subacute liver necrosis |  |
| JAUNDICE | 7745 | Perinatal jaundice from other causes |  |
|  | 7746 | Unspecified fetal and neonatal jaundice |  |
|  | 7824 | Unspecified jaundice, not of the newborn |  |
| CANCER | V580 | Radiotherapy session |  |
|  | 220 | Benign ovarian tumors |  |
|  | 1919 | Malignant brain tumors, unspecified |  |
|  | 1952 | Malignant abdominal tumors |  |
|  | 2113 | Benign colon tumors |  |
|  | 2160 | Benign tumors of the skin of the lip |  |
|  | 2273 | Benign pituitary tumors and craniopharyngeal duct (or pouch) tumors |  |
|  | 7822 | Localized superficial swellings, masses, or nodules |  |
|  | 7866 | Swelling, mass, or protrusion of the chest |  |
| GALLBLADDER STONES (CHOLELITHIASIS) | 57420 | Cholelithiasis without mention of cholecystitis, without mention of obstruction |  |
|  | 57421 | Cholelithiasis without mention of cholecystitis, with obstruction |  |
| INFECTIOUS GASTROENTERITIS | 27651 | Dehydration |  |
|  | 5362 | Persistent vomiting |  |
|  | 5550 | Regional enteritis of the small intestine |  |
|  | 5552 | Regional enteritis of the small and large intestine |  |
|  | 5559 | Regional enteritis, site unspecified |  |
|  | 78701 | Nausea with vomiting |  |
|  | 78703 | Vomiting only |  |
|  | 7877 | Abnormal stools |  |
|  | 78791 | Diarrhea |  |
|  | 7921 | Non-specific abnormal findings in stools |  |
|  | 78261 | Pallor |  |
|  | 0030. | Gastroenteritis due to Salmonella |  |
|  | 00861. | Enteritis due to Rotavirus |  |
|  | 00862. | Enteritis due to Adenovirus |  |
|  | 0090. | Infectious colitis, enteritis, and gastroenteritis |  |
|  | 0091. | Colitis, enteritis, and gastroenteritis of presumed infectious origin |  |
|  | 0092. | Febrile mucous-bloody enteritis and dehydration |  |
|  | 0093. | Diarrhea of presumed infectious origin |  |
| CYSTIC FIBROSIS | 27702 | Cystic fibrosis with pulmonary manifestations |  |
|  | 27703 | Cystic fibrosis with gastrointestinal manifestations |  |
|  | 27709 | Cystic fibrosis with other manifestations |  |
| AUTOINFLAMMATORY DISEASES | 27731 | Familial Mediterranean fever | Autoinflammatory disease/ PFAPA / Sindrome CRIA |
|  | 0879. | Recurrent fever, unspecified |  |
| OBESITY | 2511 | Other manifestations of hyperinsulinism |  |
|  | 2777 | Metabolic syndrome X |  |
|  | 27800 | Obesity, unspecified |  |
|  | 27802 | Overweight |  |
|  | 2788 | Other manifestations of overnutrition |  |
| IMMUNODEFICIENCIES | 27901 | Selective IgA deficiency |  |
|  | 27906 | Common variable immunodeficiency |  |
|  | 2793 | Unspecified immunodeficiency |  |
|  | 28733 | Congenital and hereditary thrombocytopenic purpura |  |
| ANEMIAS | 2800 | Iron deficiency anemia secondary to (chronic) blood loss |  |
|  | 2809 | Iron deficiency anemia, unspecified |  |
|  | 2818 | Anemia associated with other specified nutritional deficiency |  |
|  | 2822 | Anemia due to disorders of glutathione metabolism |  |
|  | 28241 | Sickle-cell thalassemia without crisis |  |
|  | 28260 | Sickle-cell disease, unspecified |  |
|  | 2829 | Hereditary hemolytic anemia, unspecified |  |
|  | 28310 | Non-autoimmune hemolytic anemia, unspecified |  |
|  | 2839 | Acquired hemolytic anemia, unspecified |  |
|  | 2859 | Anemia, unspecified |  |
| RHEUMATOLOGICAL DISEASES | 2870 | Allergic purpura |  |
|  | 28731 | Immune thrombocytopenic purpura |  |
|  | 7209 | Inflammatory spondylopathy, unspecified | MIS-C |
|  | 7140 | Rheumatoid arthritis |  |
|  | 71430 | Polyarticular juvenile rheumatoid arthritis, chronic or unspecified |  |
|  | 7149 | Unspecified inflammatory polyarthropathy |  |
|  | 71650 | Polyarthropathy or polyarthritis, unspecified site |  |
|  | 71665 | Monoarthritis, unspecified, hip |  |
|  | 71666 | Monoarthritis, unspecified, knee |  |
|  | 7197 | Difficulty in walking, not elsewhere classified |  |
|  | 7202 | Sacroiliitis, not elsewhere classified |  |
|  | 7290 | Fibromyalgia and diffuse unspecified non-articular rheumatism |  |
|  | 7291 | Myalgia and myositis, unspecified |  |
|  | 99590 | Systemic inflammatory response syndrome (SIRS), unspecified |  |
| ARTHTOPATHIES | 71190 | Infectious arthritis, unspecified, site unspecified |  |
|  | 71196 | Infectious arthritis, unspecified, knee |  |
|  | 71686 | Other specified arthropathies, knee |  |
|  | 71940 | Arthralgia |  |
|  | 71945 | Arthralgia, hip |  |
|  | 71949 | Arthralgia, multiple sites |  |
| HEMATOLOGY DISEASE | 20400 | Acute lymphoid leukemia, without mention of remission |  |
|  | 2841 | Pancytopenia |  |
|  | 28800 | Neutropenia, unspecified |  |
|  | 28802 | Cyclic neutropenia |  |
|  | 28804 | Neutropenia due to infection |  |
|  | 28809 | Other neutropenias |  |
|  | 2875 | Thrombocytopenia, unspecifie |  |
|  | 2883 | Acquired hemolytic anemia, unspecified |  |
|  | 2884 | Hemophagocytic syndromes |  |
| INFECTIONS | 99591 | Sepsis |  |
|  | 99592 | Severe sepsis |  |
|  | 1369 | Unspecified infectious and parasitic diseases |  |
|  | 3201 | Pneumococcal meningitis |  |
|  | 3202 | Streptococcal meningitis |  |
|  | 32089 | Meningitis due to other specified bacteria |  |
|  | 3229 | Meningitis, unspecified |  |
|  | 37601 | Orbital cellulitis |  |
|  | 3839 | Mastoiditis, unspecified |  |
|  | 075. | Infectious mononucleosis |  |
|  | 0840. | Plasmodium falciparum malaria (malignant tertian malaria) |  |
|  | 0849. | Other pernicious complications of malaria | splenic infarction |
|  | 6822 | Other phlegmons and abscesses of the trunk | Cellulitis of the lower limb |
|  | 4572 | Lymphangitis |  |
|  | 4619 | Acute sinusitis, unspecified |  |
|  | 4878 | Influenza with other manifestations |  |
|  | 5225 | Periapical abscess without sinus |  |
|  | 5283 | Cellulitis and abscess of oral soft tissues |  |
|  | 52103 | Dental caries extending to the pulp |  |
|  | 5647 | Megacolon |  |
|  | 566 | Anal and rectal abscess |  |
|  | 578 | Other specified viral exanthems |  |
|  | 5990 | Urinary tract infection, site not specified |  |
|  | 6822 | Other phlegmons and abscesses of the trunk |  |
|  | 6824 | Other phlegmons and abscesses of the hand, excluding fingers and thumb |  |
|  | 6826 | Other phlegmons and abscesses of lower limb, excluding foot |  |
|  | 6827 | Other phlegmons and abscesses of the foot, excluding toes |  |
|  | 6828 | Other phlegmons and abscesses of other specified sites |  |
|  | 684 | Impetigo |  |
|  | 68600 | Pyoderma, unspecified |  |
|  | 68100 | Phlegmon and abscess, unspecified |  |
|  | 6144 | Chronic or unspecified parametritis and pelvic cellulitis |  |
|  | 7294 | Fasciitis, unspecified |  |
|  | 740 | Herpangina |  |
|  | 7711 | Congenital cytomegalovirus infection |  |
|  | 77181 | Neonatal septicemia (sepsis) |  |
|  | 77182 | Urinary tract infections of the newborn |  |
|  | 7881 | Dysuria |  |
|  | 7908 | Viremia, unspecified |  |
|  | 73000 | Acute osteomyelitis, site unspecified |  |
|  | 73005 | Acute osteomyelitis, pelvic bones and femur |  |
|  | 73007 | Acute osteomyelitis, bones of the foot |  |
|  | 73090 | Unspecified infection of bone, site unspecified |  |
|  | 77181 | Neonatal septicemia (sepsis) |  |
|  | 77182 | Urinary tract infections of the newborn |  |
|  | 07999 | Viral infections, unspecified |  |
|  | 0785 | Cytomegalic disease |  |
|  | 0790 | Adenovirus infections in diseases classified elsewhere and of unspecified site |  |
|  | 0793 | Rhinovirus infections in diseases classified elsewhere and of unspecified site |  |
|  | 0796 | Respiratory syncytial virus |  |
|  | 07989 | Other specified viral infections |  |
|  | 78841 | Pollakiuria |  |
|  | 01090 | Primary tuberculosis infection, unspecified |  |
|  | 0270 | Listeriosis |  |
|  | 035 | Erysipelas |  |
|  | 0380 | Streptococcal septicemia |  |
|  | 03842 | Escherichia coli (E. coli) septicemia |  |
|  | 0419 | Bacterial infection, unspecified, in diseases classified elsewhere and of unspecified site |  |
|  | 0479 | Unspecified viral meningitis |  |
|  | 0542 | Herpetic gingivostomatitis |  |
|  | 0528 | Chickenpox with unspecified complications |  |
|  | 05479. | Herpes simplex with other specified complications |  |
|  | 0578 | Other specified viral exanthems | Sixth disease |
|  | 0579 | Other unspecified viral exanthems |  |
|  | 0020 | Typhoid fever |  |
|  | 0022 | Paratyphoid B |  |
|  | 0029 | Unspecified paratyphoid |  |
|  | 0039 | Unspecified salmonella infections |  |
|  | 00867 | Enteritis due to Enterovirus NIA |  |
|  | 00801 | Intestinal infection due to enteropathogenic Escherichia coli |  |
|  | 00843 | Intestinal infection due to Campylobacter |  |
|  | 00845 | Intestinal infection due to Clostridium difficile |  |
|  | 13101 | Vulvovaginitis |  |
|  | 0880 | Bartonellosis |  |
|  | 0900 | Early congenital syphilis with symptoms |  |
|  | 0793 | Rhinovirus infections in diseases classified elsewhere and of unspecified site |  |
| NEUROPSYCHIATRY | 29384 | Anxiety disorders in diseases classified elsewhere |  |
|  | 29610 | Mania, recurrent episode, unspecified |  |
|  | 29690 | Unspecified mood disorder, episodic |  |
|  | 29900 | Childhood autism, active state |  |
|  | 30023 | Social phobia |  |
|  | 3003 | Obsessive-compulsive disorder |  |
|  | 3004 | Dysthymic disorder |  |
|  | 30753 | Rumination disorder |  |
|  | 3077 | Encopresis |  |
|  | 3082 | Predominantly psychomotor disorders |  |
|  | 3154 | Developmental coordination disorder |  |
|  | 3158 | Other specified developmental delays |  |
|  | 3159 | Unspecified developmental delay |  |
|  | 3181 | Severe intellectual disability |  |
|  | 3440 | Quadriplegia and tetraparesis |  |
|  | 3439 | Cerebral palsy, unspecified |  |
|  | 43810 | Speech and language deficits, unspecified |  |
|  | 5649 | Unspecified functional disorder |  |
|  | V6284 | Suicidal ideation |  |
|  | V403 | Other behavioral problems |  |
|  | V691 | Inappropriate diet and eating habits |  |
|  | V409 | Unspecified mental or behavioral problem |  |
|  | 311 | Depressive disorder, not elsewhere classified |  |
|  | 7812 | Gait disorders |  |
|  | 7818 | Neurological syndromes of inattention |  |
| MALNUTRITION | 3071 | Anorexia Nervosa |  |
|  | 30750 | Eating disorder, unspecified |  |
|  | 30759 | Other eating disorders | hyponutrition |
|  | 73300 | Osteoporosis, unspecified |  |
|  | 7793 | Feeding problems in newborn |  |
|  | 7830 | Anorexia |  |
|  | 7833 | Feeding difficulties and errors in food administration |  |
|  | 78321 | Weight loss |  |
|  | 262 | Other severe protein-calorie malnutrition |  |
|  | 267 | Ascorbic acid deficiency |  |
| ENCEPHALITIS-CEREBELLITIS-DEGENERATIVE DISEASES | 1390 | Sequelae of viral encephalitis |  |
|  | 32301 | Encephalitis and encephalomyelitis in viral diseases classified elsewhere |  |
|  | 32361 | Acute disseminated encephalomyelitis (ADEM) |  |
|  | 32381 | Other causes of encephalitis and encephalomyelitis |  |
|  | 3239 | Unspecified causes of encephalitis, myelitis, and encephalomyelitis |  |
|  | 3335 | Other forms of chorea |  |
|  | 3344 | Cerebellar ataxia in diseases classified elsewhere |  |
|  | 34830 | Encephalopathy, unspecified |  |
|  | 3510 | Peripheral facial palsy |  |
|  | 3518 | Other disorders of facial nerve |  |
|  | 3519 | Facial nerve disorders, unspecified |  |
|  | 3570 | Acute infective polyneuritis |  |
|  | 43884 | Ataxia |  |
|  | 0463 | Progressive multifocal leukoencephalopathy |  |
|  | 048 | Other enterovirus diseases of central nervous system |  |
|  | 37730 | Optic neuritis, unspecified |  |
|  | V291 | Observation for suspected neurological condition |  |
| SEIZURES | 34500 | Generalized non-convulsive epilepsy, without mention of intractable epilepsy |  |
|  | 30747 | Other sleep or arousal phase disorders |  |
|  | 34510 | Generalized convulsive epilepsy, without mention of intractable epilepsy |  |
|  | 3453 | Epileptic status epilepticus (generalized convulsive status) |  |
|  | 34550 | Partial epilepsy, without mention of impairment of consciousness and without mention of intractable epilepsy |  |
|  | 34580 | Other forms of epilepsy, without mention of intractable epilepsy | West syndrome |
|  | 34590 | Epilepsy, unspecified, without mention of intractable epilepsy |  |
|  | 34591 | Epilepsy, unspecified, with intractable epilepsy |  |
|  | 78031 | Febrile seizures (simple), unspecified |  |
|  | 78032 | Complex febrile seizures |  |
|  | 78039 | Other convulsions |  |
|  | 36100 | Retinal detachment with retinal break, unspecified |  |
|  | 36234 | Transient retinal artery occlusion |  |
|  | 36410 | Chronic iridocyclitis, unspecified | Chronic uveitis |
|  | 37230 | Conjunctivitis, unspecified |  |
|  | 3659 | Glaucoma, unspecified |  |
|  | 3682 | Diplopia |  |
|  | 4387 | Visual disturbances |  |
|  | 3789 | Unspecified disorders of eye movements |  |
| OTORHINOLARYNGOLOGY | 38200 | Acute suppurative otitis media without spontaneous rupture of tympanic membrane |  |
|  | 38100 | Acute nonsuppurative otitis media, unspecified |  |
|  | 38101 | Acute serous otitis media |  |
|  | 38102 | Acute mucoid otitis media |  |
|  | 3824 | Suppurative otitis media, unspecified |  |
|  | 3829 | Otitis media, unspecified |  |
|  | 38300 | Acute mastoiditis without complications |  |
|  | 38301 | Subperiosteal abscess of mastoid |  |
|  | 38302 | Acute mastoiditis with other complications | Vestibular abscess/Gradenigo's syndrome |
|  | 43885 | Vertigo |  |
|  | 4461 | Acute febrile mucocutaneous lymph node syndrome (MCLS) |  |
|  | 47875 | Laryngeal spasm |  |
|  | 5272 | Sialadenitis |  |
|  | 5282 | Oral aphthosis |  |
|  | 52800 | Stomatitis and mucositis, unspecified |  |
|  | 683 | Acute lymphadenitis |  |
|  | 287 | Control of hemorrhage after tonsillectomy and adenoidectomy |  |
|  | 2893 | Unspecified lymphadenitis, except mesenteric |  |
|  | 7847 | Epistaxis |  |
|  | 7856 | Lymphadenomegaly |  |
|  | 7861 | Stridor |  |
| COAGULOPATHIES | 2864 | Von Willebrand disease |  |
|  | 325 | Phlebitis and thrombophlebitis of intracranial venous sinuses |  |
|  | 452 | Portal vein thrombosis |  |
|  | 41511 | Iatrogenic pulmonary embolism and infarction |  |
|  | 45189 | Phlebitis and thrombophlebitis of other sites |  |
| CARDIAC DISEASE | 42090 | Acute pericarditis, unspecified |  |
|  | 4275 | Cardiac arrest |  |
| ESOPHAGITIS AND GASTRITIS | 43882 | Dysphagia |  |
|  | 53010 | Esophagitis, unspecified |  |
|  | 53011 | Reflux esophagitis |  |
|  | 53012 | Acute esophagitis |  |
|  | 53019 | Other esophagitis |  |
|  | 53081 | Esophageal reflux |  |
|  | 53500 | Acute gastritis without mention of hemorrhage |  |
|  | 53510 | Atrophic gastritis without mention of hemorrhage |  |
|  | 53540 | Other specified gastritis without mention of hemorrhage |  |
|  | 53550 | Unspecified gastritis and gastroduodenitis without mention of hemorrhage |  |
|  | 7872 | Dysphagia |  |
| PNEUMONIA-BRONCHITIS-UPPER RESPIRATORY TRACT INFECTIONS | 4660 | Acute bronchitis |  |
|  | 46619 | Acute bronchiolitis due to other infectious organisms |  |
|  | 46611 | Acute bronchiolitis due to respiratory syncytial virus (RSV) |  |
|  | 4801 | Pneumonia due to respiratory syncytial virus |  |
|  | 4802 | Pneumonia due to parainfluenza virus |  |
|  | 4809 | Viral pneumonia, unspecified |  |
|  | 481 | Pneumococcal pneumonia (Pneumonia due to Streptococcus pneumoniae) |  |
|  | 4821 | Pneumonia due to Pseudomonas |  |
|  | 4829 | Bacterial pneumonia, unspecified |  |
|  | 4830 | Pneumonia due to Mycoplasma pneumoniae |  |
|  | 485 | Bronchopneumonia, unspecified |  |
|  | 486 | Pneumonia, unspecified organism |  |
|  | 4870 | Influenza with pneumonia |  |
|  | 490 | Bronchitis, unspecified whether acute or chronic |  |
|  | 49120 | Chronic obstructive bronchitis without exacerbation |  |
|  | 49121 | Chronic obstructive bronchitis with acute exacerbation |  |
|  | 4941 | Bronchiectasis with exacerbation |  |
|  | 5070 | Aspiration pneumonia due to food or vomit |  |
|  | 5109 | Empyema without mention of fistula |  |
|  | 5110 | Pleurisy without mention of effusion or active tuberculosis |  |
|  | 51881 | Respiratory failure |  |
|  | 48231 | Pneumonia due to Streptococcus group A |  |
|  | 49121 | Chronic obstructive bronchitis with acute exacerbation |  |
|  | 510 | Empyema |  |
|  | 51884 | Acute and chronic respiratory failure |  |
|  | 51889 | Other lung diseases, not elsewhere classified | Pulmonary consolidation |
|  | 7705 | Other and unspecified atelectasis of newborn |  |
|  | 77083 | Cyanotic spells of newborn |  |
|  | 7825 | Cyanosis |  |
|  | 78600 | Unspecified respiratory abnormalities (Dyspnea/Respiratory distress) |  |
|  | 78603 | Apnea |  |
|  | 78609 | Other dyspnea and respiratory abnormalities (Respiratory difficulty) |  |
|  | 7862 | Cough |  |
|  | 7867 | Abnormal chest sounds |  |
| PHARYNGITIS-ADENOSITIS-TONSILLITIS | 462 | Acute pharyngitis |  |
|  | 463 | Acute tonsillitis |  |
|  | 46400 | Acute laryngitis without mention of obstruction |  |
|  | 46410 | Acute tracheitis without mention of obstruction |  |
|  | 4659 | Acute upper respiratory infection, unspecified site |  |
|  | 47402 | Chronic tonsillitis and adenoiditis |  |
|  | 47821 | Cellulitis of pharynx or nasopharynx |  |
|  | 47824 | Retropharyngeal abscess |  |
| ABDOMINAL PAIN | 78900 | Abdominal pain, unspecified location |  |
|  | 2892 | Mesenteric lymphadenitis, unspecified |  |
|  | 78900 | Abdominal pain, unspecified location |  |
|  | 78901 | Right upper quadrant abdominal pain |  |
|  | 78903 | Right lower quadrant abdominal pain |  |
|  | 78904 | Left lower quadrant abdominal pain |  |
|  | 78905 | Periumbilical abdominal pain |  |
|  | 78907 | Generalized abdominal pain |  |
| COMA | 30740 | Nonorganic sleep disorder, unspecified |  |
|  | 7802 | Syncope and collapse |  |
|  | 78002 | Temporary (or transient) loss of consciousness |  |
|  | 78097 | Altered mental status |  |
|  | 78009 | Other disturbances of consciousness |  |
|  | 8500 | Concussion without loss of consciousness |  |
|  | 8502 | Concussion with moderate loss of consciousness |  |
|  | 8504 | Concussion with prolonged loss of consciousness, without return to pre-existing level of consciousness |  |
| TRAUMA | 4230 | Hemopericardium |  |
|  | 431 | Cerebral hemorrhage |  |
|  | 4590 | Hemorrhage, unspecified |  |
|  | 938 | Foreign body in digestive tract, unspecified site |  |
|  | 1435 | Repair of retinal tear by unspecified type of photocoagulation |  |
|  | 80000 | Closed fracture of cranial vault without mention of intracranial injury, consciousness state unspecified |  |
|  | 80016 | Closed fracture of cranial vault without mention of intracranial injury, without loss of consciousness |  |
|  | 8020 | Closed fracture of nasal bones |  |
|  | 8021 | Open fracture of nasal bones |  |
|  | 80499 | Open fractures involving skull or face with other bones, with other and unspecified intracranial injury, with unspecified cerebral concussion |  |
|  | 80620 | Closed fracture of T1–T6 segment with unspecified spinal cord injury |  |
|  | 81220 | Closed fracture of unspecified part of humerus |  |
|  | 81250 | Open fracture of unspecified part of distal end of humerus |  |
|  | 81393 | Closed fracture of radius and ulna |  |
|  | 85102 | Cerebral cortex contusion without mention of open intracranial wound, with brief [less than one hour] loss of consciousness |  |
|  | 85206 | Traumatic subarachnoid hemorrhage without mention of open intracranial wound, with unspecified duration of loss of consciousness |  |
|  | 85400 | Other and unspecified intracranial injuries without open intracranial wound, consciousness state unspecified |  |
|  | 85401 | Other and unspecified intracranial injuries without open intracranial wound, without loss of consciousness |  |
|  | 85412 | Other and unspecified intracranial injuries with open intracranial wound, with brief [less than one hour] loss of consciousness |  |
|  | 86384 | Injury of pancreas, multiple and unspecified sites |  |
|  | 86502 | Splenic injury without mention of open wound into cavity, capsular laceration without major parenchymal disruption |  |
|  | 86509 | Splenic injury without mention of open wound into cavity, other |  |
|  | 86800 | Injury of other intra-abdominal organs without open wound into cavity, unspecified intra-abdominal organ |  |
|  | 8690 | Internal injury of unspecified or ill-defined organs without open wound into cavity |  |
|  | 83230 | Pathological dislocation of elbow |  |
|  | 73310 | Pathological fracture, unspecified site |  |
|  | 73311 | Pathological fracture of humerus |  |
|  | 73313 | Pathological fracture of vertebrae |  |
|  | 73315 | Pathological fracture of other specified parts of femur |  |
|  | 9352 | Foreign body in stomach |  |
|  | 94504 | Burn of unspecified degree, leg |  |
|  | 94811 | Burn involving 10%–19% of body surface, third-degree burns involving 10%–19% of body surface |  |
|  | 9639 | Poisoning by unspecified systemic agents |  |
|  | 9941 | Drowning |  |
| SYNDROMES | 5564 | Familial Adenomatous Polyposis |  |
|  | 23771 | Neurofibromatosis type I (Von Recklinghausen disease) |  |
|  | 7572 | Dermatoglyphic anomalies | sindrome SAM (Severe dermatitis, multiple Allergies and Metabolica wasting syndrome) |
|  | 7560 | Anomalies of skull and facial bones |  |
|  | 7587 | Klinefelter syndrome |  |
|  | 7595 | Tuberous sclerosis |  |
|  | 7597 | Multiple congenital anomalies, so described |  |
|  | 7598 | Other specified congenital anomalies |  |
|  | 75981 | Prader-Willi syndrome |  |
|  | 73681 | Acquired leg length discrepancy |  |
|  | 75651 | Osteogenesis imperfecta |  |
|  | 75889 | Other manifestations due to chromosomal anomalies |  |
|  | 75981 | Prader-Willi syndrome |  |
|  | 75989 | Other specified anomalies | CHARGE syndrome/Silver-Russell syndrome |
|  | V293 | Observation for suspected genetic or metabolic disease |  |
| COVID-19 | 04311 | Confirmed COVID-19 disease, virus identified |  |
|  | 04321 | Paucisymptomatic COVID-19 disease, virus identified |  |
|  | 48041 | COVID-19 pneumonia, virus identified |  |
|  | 4803 | Pneumonia due to SARS-associated coronavirus |  |
|  | 51891 | Acute Respiratory Distress Syndrome (ARDS) in COVID-19, virus identified – Excludes: ARDS or respiratory failure following trauma or surgical procedures, other forms of respiratory distress |  |
|  | 51892 | Acute Respiratory Distress Syndrome (ARDS) in COVID-19, virus not identified – COVID-19 diagnosed on clinical-epidemiological basis, probable or suspected COVID-19 | ARDS in COVID-19 |
| ALLERGIES | 49302 | Extrinsic asthma with (acute) exacerbation |  |
|  | 49322 | Chronic obstructive asthma with acute exacerbation |  |
|  | 49382 | Cough variant asthma |  |
|  | 49390 | Asthma, unspecified type | Asthmatic bronchitis |
|  | 49392 | Asthma, unspecified type with acute exacerbation |  |
|  | 51911 | Acute bronchospasm |  |
|  | 5583 | Allergic gastroenteritis and colitis |  |
|  | 5589 | Noninfectious gastroenteritis and colitis |  |
|  | 78607 | Wheezing |  |
|  | 9950 | Other anaphylactic shock, not elsewhere classified |  |
|  | 9953 | Allergy, unspecified, not elsewhere classified |  |
|  | V1502 | Personal history of allergy to dairy products |  |
|  | V1503 | Personal history of allergy to eggs |  |
| SURGERY | 53642 | Mechanical complication of gastrostomy |  |
|  | 60824 | Torsion of epididymal appendix |  |
|  | 4523 | Anal or rectal polyp |  |
| ABDOMINAL MALFORMATIONS | 55010 | Unilateral or unspecified inguinal hernia, with obstruction, without mention of gangrene |  |
|  | 56949 | Other specified disorders of the rectum and anus |  |
|  | 7513 | Hirschsprung's disease and other congenital functional disorders of the colon |  |
| INTESTINAL OBSTRUCTIONS | 541 | Appendicitis, unspecified |  |
|  | 542 | Other forms of appendicitis |  |
|  | 5401 | Acute appendicitis with peritoneal abscess |  |
|  | 5409 | Acute appendicitis without mention of peritonitis, perforation, or rupture |  |
|  | 5600 | Intussusception |  |
|  | 5602 | Volvulus |  |
|  | 5609 | Unspecified intestinal obstruction |  |
|  | 56030 | Intestinal impaction, unspecified |  |
|  | 56089 | Other specified intestinal obstruction |  |
| NEPHROLOGICAL DISEASES | 5809 | Acute glomerulonephritis, with unspecified histological lesions |  |
|  | 5819 | Nephrotic syndrome, with unspecified histological lesions |  |
|  | 5849 | Acute renal failure, unspecified |  |
|  | 5889 | Unspecified diseases caused by reduced renal function |  |
|  | 586 | Renal failure, unspecified |  |
|  | 59010 | Acute pyelonephritis, without medullary necrosis |  |
|  | 59080 | Pyelonephritis, unspecified | Urinary tract sepsis |
|  | 28311 | Hemolytic uremic syndrome |  |
|  | 5997 | Hematuria |  |
|  | 7885 | Oliguria and anuria |  |
| ORTHOPEDICS DISEASES | 1026 | Bone and joint injuries |  |
|  | 7218 | Other disorders of the vertebral column |  |
|  | 7231 | Cervicalgia (Neck pain) |  |
|  | 7242 | Lumbalgia (Lower back pain) |  |
|  | 7295 | Pain in the limbs |  |
|  | 7326 | Other juvenile osteochondroses |  |
|  | 71925 | Villonnodular synovitis, hip |  |
|  | 71966 | Other disorders of the joints, knee |  |
|  | 73743 | Scoliosis associated with other morbid manifestations |  |
|  | 73739 | Other kyphoscoliosis and scoliosis |  |
|  | 78199 | Other symptoms of the nervous and musculoskeletal system |  |
|  | 92310 | Contusion of the forearm |  |
| NEUROMUSCOLAR DISORDER – MUSCOLAR ATOPHY | 72887 | Muscular weakness (Generalized) | Asthenia |
|  | 7810 | Abnormal involuntary movements |  |
|  | 72888 | Rhabdomyolysis |  |
|  | 3332 | Myoclonus |  |
| EDEMA | 72981 | Edema of the limbs |  |
|  | 7823 | Edema |  |
| CYSTS DERIVE FROM BRANCHIAL ARCHES | 74441 | Branchial cyst or fistula |  |
|  | 74900 | Cleft palate, unspecified |  |
|  | 74920 | Cleft palate with cleft lip, unspecified |  |
| LIVER MLAFORMATIONS | 74760 | Anomalies of the peripheral vascular system, unspecified site |  |
|  | 75160 | Unspecified anomalies of the gallbladder, bile ducts, and liver |  |
|  | 75169 | Other anomalies of the gallbladder, bile ducts, and liver |  |
|  | 5768 | Other specified biliary tract disorders |  |
| UROGENITAL MALFORMATIONS | 591 | Hydronephrosis |  |
|  | 59654 | Neurogenic bladder SAI |  |
|  | 5939 | Unspecified pathology of the kidney and ureter |  |
|  | 605 | Exuberant foreskin and phimosis |  |
|  | 75310 | Unspecified renal cystic disease |  |
| PREMATURE | 76500 | Extreme immaturity of the neonate, unspecified weight |  |
|  | 76510 | Disorders related to other preterm neonates, unspecified weight |  |
| ENDOCRINOLOGY DISEASE | 78340 | Defect in normal physiological development, unspecified | Failure to thrive |
|  | 78341 | Failure of stature-weight growth |  |
|  | 78342 | Delayed milestones |  |
|  | 78343 | Short stature |  |
|  | 243 | Congenital hypothyroidism |  |
|  | 2449 | Unspecified hypothyroidism |  |
|  | 2532 | Anterior panhypopituitarism |  |
|  | 2554 | corticoadrenal insufficiency |  |
|  | 2551 | Unspecified hyperaldosteronism |  |
|  | 6260 | Absence of menstruation |  |
|  | 6262 | Heavy or frequent menstruation |  |
|  | 6269 | Menstrual disorders and other abnormal genital tract bleeding in females, unspecified |  |
|  | 2420 | Diffuse toxic goiter without thyrotoxic crisis |  |
|  | 2591 | Precocious sexual development and precocious puberty, not classified elsewhere |  |
|  | 2572 | Hypogonadism |  |
| CHEST PAIN | 78659 | Other chest pain |  |
| DIABETES | 25000 | Diabetes Mellitus, type II or unspecified, not defined as decompensated, without mention of complications |  |
|  | 25001 | Diabetes Mellitus, type I (Juvenile Diabetes), not defined as decompensated, without mention of complications |  |
|  | 25002 | Diabetes Mellitus, type II or unspecified, decompensated, without mention of complications |  |
|  | 25003 | Diabetes Mellitus, type I (Juvenile Diabetes), decompensated |  |
|  | 25010 | Diabetes with Ketoacidosis, type II or unspecified, not defined as decompensated |  |
|  | 25011 | Diabetes with Ketoacidosis, type I (Juvenile Diabetes), not defined as decompensated |  |
|  | 25013 | Diabetes with Ketoacidosis, type I (Juvenile Diabetes), decompensated |  |
|  | 25020 | Diabetes with Hyperosmolarity, type II or unspecified, not defined as decompensated |  |
|  | 2535 | Diabetes Insipidus |  |
| DRUG POISON | E8542 | Accidental intoxication from other psychotropic agents. From psychostimulants |  |
|  |  | Accidental intoxication from other psychotropic agents. From psychedelics (hallucinogens) |  |
|  | E8589 | Accidental intoxication from other drugs. From unspecified drugs |  |
|  | E8614 | Accidental poisoning from detergents and polishes, disinfectants, varnishes, and paints. Disinfectants |  |
|  | E8620 | Accidental poisoning from petroleum products, other solvents, and their vapors, not classified elsewhere. Petroleum solvents |  |
|  | E8664 | Accidental poisoning from other unspecified solid and liquid substances. Other metal compounds and their fumes |  |
|  | E9806 | Poisoning from solid or liquid substances, unspecified whether inflicted accidentally or intentionally. Corrosive and caustic substances |  |
|  | 305 | Drug abuse without dependence |  |
|  | 9095 | Aftereffects of adverse effects from drugs, medicines, or other biological products |  |
|  | 9699 | Poisoning from unspecified psychotropic substances |  |
|  | 9719 | Poisoning from unspecified drugs primarily affecting the autonomic nervous system |  |
|  | 9779 | Poisoning from unspecified drug or medication |  |
|  | 9830 | Toxic effects of aromatic corrosive substances, acids, and caustic alkalis |  |
| DERMATOLOGICAL DISEASE | 7821 | Rash and other unspecified skin eruptions |  |
|  | 6869 | Unspecified localized skin and subcutaneous tissue infections |  |
|  | 6918 | Other atopic dermatitis and related conditions |  |
|  | 6929 | Dermatitis from unspecified causes |  |
|  | 6950 | Toxic erythema |  |
|  | 6951 | Multiform or polymorphic erythema |  |
|  | 6959 | Unspecified erythematous conditions |  |
|  | 6989 | Unspecified pruritic conditions |  |
|  | 7041 | Hirsutism |  |
|  | 7048 | Other specified diseases of the hair and hair follicles |  |
|  | 7062 | Sebaceous cyst |  |
|  | 22800 | Hemangioma of unspecified location |  |
|  | 22809 | Hemangioma of other sites |  |
|  | 7089 | Urticaria, unspecified |  |
| FUNCTIONAL GASTROINTESTINAL DISORDER | 3064 | Gastrointestinal dysfunctions |  |
|  | 56400 | Constipation, unspecified |  |
|  | 5641 | Irritable bowel syndrome |  |
|  | 7873 | Flatulence, belching, and gas colic |  |
| METABOLIC DISEASES | 2512 | Unspecified hypoglycemia |  |
|  | 2774 | Primary disorders of bilirubin metabolism |  |
| HYPERCOLESTEROLEMIA | 2720 | Hypercholesterolemia |  |
| CEREBRAL MALFORMATIONS | 3314 | Obstructive hydrocephalus |  |
|  | 3480 | Cerebral cysts |  |
|  | 7423 | Congenital hydrocephalus |  |
|  | 7422 | Congenital deformities with reduction of the brain |  |
| HYPERTENSION | 3482 | Benign idiopathic intracranial hypertension |  |
|  | 99791 | Hypertension |  |
| ARRHYTHMIAS | 4270 | *Paroxysmal supraventricular tachycardia* |  |
|  | 7850 | Tachycardia, unspecified |  |
| LYMPHEDEMA | 4571 | Other lymphedemas |  |
| PULMONARY AND UPPER RESPIRATORY TRACT MALFORMATIONS | 5128 | Other spontaneous pneumothoraxes |  |
|  | 8600 | Pneumothorax without mention of open chest wound |  |
| INFLAMMATORY BOWEL DISEASE | 5562 | Ulcerative proctitis (chronic) |  |
|  | 5565 | Ulcerative colitis localized to the colon |  |
|  | 5566 | *Ulcerative pancolitis* |  |
|  | 5569 | Ulcerative colitis, unspecified |  |
|  | 5781 | Melena and rectal bleeding |  |
|  | 5650 | Anal fissure |  |
| CHOLECYSTITIS | 5750 | Acute cholecystitis |  |
|  | 5753 | Hydrops of the gallbladder |  |
|  | 5760 | Post-cholecystectomy syndrome |  |
|  | 57510 | Cholecystitis, unspecified |  |
| PANCREATIC INSUFFICIENCY | 5770 | Acute pancreatitis |  |
| CELIAC DISEASE | 5790 | Celiac disease |  |
| KIDNEY STONES (NEPHROLITHIASIS) | 5920 | Kidney stones |  |
|  | 7880 | Renal colic |  |
| FEVER | 7806 | Fever |  |
| HEADACHE | 7840 | Headache |  |
|  | 34600 | Classic migraine without mention of intractable migraine |  |
|  | 34690 | Unspecified migraine without mention of intractable migraine |  |
| ASCITES | 7895 | Ascites |  |
| ELEVATED TRANSAMINASES | 7904 | Non-specific increase in transaminase or lactate dehydrogenase (LDH) levels |  |
| ACIDOSIS | 7757 | Late metabolic acidosis of the newborn |  |
| BILIARY ATRESIA | 75161 | Biliary atresia |  |
|  | 4588 | Other specified hypotension |  |
| CARDIAC MALFOMATIONS | 4299 | Unspecified heart disease |  |
|  | 3969 | Diseases of the mitral valve and aortic valve, unspecified |  |
| OTHERS | 7905 | Other non-specific abnormal serum enzyme values |  |
|  | 9999 | Other and unspecified complications of medical care, not classified elsewhere |  |
|  | 60490 | Unspecified orchitis and epididymitis |  |
|  | 78099 | Other general symptoms |  |
|  | 2776 | Other deficiencies of circulating enzymes | Angioedema |
|  | V6759 | Other follow-up visit |  |
|  | V714 | Observation following another incident |  |
|  | V7181 | Observation for suspected abuse and neglect |  |
|  | V441 | Presence of gastrostomy |  |
|  | V5881 | Placement and positioning of vascular catheter |  |
|  | 1027 | Other manifestations |  |

***Supplemental table 6.*** Classification into diagnostic group based on the diagnosis linked to ICD-9-CM code.
